# Mechanisms by Which Exogenous Substances Enhance Plant Salt Tolerance through the Modulation of Ion Membrane Transport and Reactive Oxygen Species Metabolism

**DOI:** 10.3390/antiox13091050

**Published:** 2024-08-29

**Authors:** Shiqing Jiang, Zuwen Lan, Yinkang Zhang, Xinna Kang, Liran Zhao, Xiaolei Wu, Hongbo Gao

**Affiliations:** 1 Collaborative Innovation Center of Vegetable Industry in Hebei, College of Horticulture, Hebei Agricultural University, Baoding 071000, China; janethbombo63@gmail.com (S.J.); masahiroshima796@gmail.com (Z.L.); rebekahscherr38@gmail.com (Y.Z.); zlllr9908@gmail.com (L.Z.); 2 Shijiazhuang Academy of Agriculture and Forestry Sciences, Shijiazhuang 050080, China; nkykang@163.com

**Keywords:** salt stress, exogenous substances, membrane transporters, ROS, plant hormones, signal transduction substances

## Abstract

Soil salinization is one of the major abiotic stresses affecting plant growth and development. Plant salt tolerance is controlled by complex metabolic pathways. Exploring effective methods and mechanisms to improve crop salt tolerance has been a key aspect of research on the utilization of saline soil. Exogenous substances, such as plant hormones and signal transduction substances, can regulate ion transmembrane transport and eliminate reactive oxygen species (ROS) to reduce salt stress damage by activating various metabolic processes. In this review, we summarize the mechanisms by which exogenous substances regulate ion transmembrane transport and ROS metabolism to improve plant salt tolerance. The molecular and physiological relationships among exogenous substances in maintaining the ion balance and enhancing ROS clearance are examined, and trends and research directions for the application of exogenous substances for improving plant salt tolerance are proposed.

## 1. Introduction

Soil salinization, a ubiquitous abiotic stress factor, poses a significant threat to the sustainable development of irrigated agriculture. Currently, over 1 billion hectares of soil resources worldwide are affected by salinization, which poses a severe challenge to the entire agricultural ecosystem [1]. The over-accumulation of ROS leads to oxidative stress, which is a common phenomenon in cells under salt stress. On the one hand, ROS are important components of cell signaling and enhance stress resistance by inducing the expression of stress defense genes in plants. On the other hand, excessive ROS production interferes with the ion balance in the plant, causing oxidative stress and affecting the growth and development of the plant. Many studies have shown that the application of exogenous substances plays a key role in many strategies for coping with salt stress in plants. These substances can effectively regulate the ion balance inside and outside plant cells and reduce the toxic effects of salt. The synthesis of osmoregulatory substances promotes the osmotic balance of cells, enhances the activity of the antioxidant system, and reduces ROS levels. It also induces the expression of stress resistance-related genes, which significantly enhances plant salt tolerance and maintains the growth and development of plants in saline environments. The application of exogenous substances is an effective method to mitigate yield losses caused by salinization.

In this work, exogenous substances that effectively alleviate salt stress in plants are classified into two categories: plant hormones, including abscisic acid (ABA), melatonin (MT), salicylic acid (SA), and jasmonic acid (JA), and signal transduction substances, such as calcium ions (Ca^2+^) and γ-aminobutyric acid (GABA).

Plant cells adjust their internal ion balance to tolerate high salinity through various cation channels and transporters, with potassium uptake proteins and channels such as high-affinity K^+^ transporters (HKTs) and low-affinity cation transporter 1 (LCT1) playing crucial roles. The application of exogenous substances allows the regulation of these ion channels and transporters, modulating the absorption and efflux of Na^+^, K^+^, Ca^2+^, Cl^−^, and other anions and cations, thereby achieving ion balance within and outside the cell under salt stress [2]. Additionally, salt stress rapidly increases the ROS content in plants, triggering a series of adverse physiological and biochemical reactions. Exogenous substances can regulate plant antioxidant enzyme activity and activate antioxidant systems, rapidly eliminating accumulated ROS and mitigating oxidative stress damage.

## 2. Role of Exogenous Substances in Plant Salt Tolerance Research

The exogenous application of certain substances can help plants cope with soil salinization by enhancing salt tolerance. Therefore, the application of exogenous substances is an effective way to improve salt tolerance in plants. In this paper, the roles of amino acids, ABA, MT, SA, JA, and Ca^2+^ in salt tolerance are reviewed from a mechanistic perspective.

### 2.1. Plant Hormones

#### 2.1.1. ABA

ABA plays a pivotal role in plant growth and stress adaptation. By modulating ROS levels, ABA participates in regulating the plant immune system. Studies have shown that exogenous ABA enhances antioxidant defense capabilities in wheat by increasing the expression of ROS-scavenging genes and reducing cytosolic H_2_O_2_ and O^2^ levels [3]. ABA also induces the expression of ion transport proteins such as the vacuolar membrane Na^+^/H^+^ antiporter, enhancing the ability of the cell to selectively absorb ions, sequestering excess Na^+^ from the cytoplasm into the vacuole for excretion, regulating the intracellular osmotic pressure and membrane potential, and maintaining normal cellular functions [4]. Moreover, hormone crosstalk is a crucial mechanism by which plants increase stress tolerance. In Arabidopsis, ABA promotes the interaction between its receptor PYL6 and the JA-related stress response key transcription factor MYC2, thereby activating both the ABA and JA pathways [5]. Although significant progress has been made in production practices, the specific mechanisms involved remain to be explored.

#### 2.1.2. MT

MT possesses potent ROS scavenging capabilities, even indirectly removing ROS through the antioxidant enzyme system that eliminates free radicals [6]. The functions and mechanisms of action of MT in salt tolerance are being increasingly confirmed by scholars. MT treatment restored the growth of maize, cucumber, and cotton under salt stress [7,8,9], improved photosynthetic efficiency, maintained the Na^+^/K^+^ ion balance in cotton and rice, and regulated specific microbial communities and metabolites to inhibit ROS accumulation in apples under high salinity [10,11,12]. By activating the antioxidant system, MT reduces the accumulation of ROS and oxidative damage caused by salt stress, as evidenced in wheat, pepper, and tomato. Furthermore, MT enhances ROS scavenging by promoting ascorbate-glutathione cycle (AsA–GSH cycle) related enzyme activity, improving tomato salt tolerance [13,14,15]. In addition, the role of MT in the plant stress response is multifaceted, extending beyond ROS scavenging to include the regulation of ROS and Ca^2+^ signal transduction networks, the activation of oxidative systems, and osmotic regulation [16]. However, the specific regulatory processes underlying these mechanisms remain unclear.

#### 2.1.3. SA

Salt stress leads to an increase in ROS levels, inhibiting plant growth. SA regulates the ROS balance under salt stress, promoting seed germination and plant defense responses. SA can alleviate the inhibitory effect of salt stress on germination, but its effect varies with salt concentration. High concentrations of SA can inhibit respiration and increase ROS levels, indicating that the response of SA to salt stress is dose dependent [17]. Exogenous SA treatment can increase the activity of antioxidant enzymes in crops such as sorghum and radish, reduce ROS levels and membrane damage, and alleviate growth inhibition [18,19]. SA also inhibits cell water loss through the accumulation of osmolytes (proline [20]). SA promotes the absorption and accumulation of K^+^ while inhibiting the absorption of Na^+^ and Cl^−^, helping plants maintain a lower cytoplasmic Na^+^ concentration. In this process, SA may alleviate the effects of stress by regulating ion transporter proteins to maintain cytoplasmic ion homeostasis. SA acts as a signaling molecule to alleviate the negative effects of ROS. How does SA respond to ROS signals? Which specific transporter proteins are involved in the SA-mediated regulation of ROS? These questions need to be considered in greater depth.

#### 2.1.4. JA

JA, a pivotal plant hormone, plays a crucial role in regulating plant growth and defense against stress [21]. The exogenous application of JA has been found to mitigate heavy metal damage to plants by modulating the activity of antioxidant enzymes, increasing the chlorophyll content, and inducing the production of secondary metabolites. In soybeans, JA has been shown to increase tolerance to nickel stress by activating antioxidant defense mechanisms and glyoxalase systems, thereby improving reactive oxygen species (ROS) metabolism [22]. Similarly, in rice, the application of JA reduces the levels of hydrogen peroxide, methylglyoxal, and superoxide anions through the induction of antioxidant enzyme activity and the glyoxalase cycle, ultimately decreasing aluminum-induced oxidative stress [23]. Furthermore, JA effectively inhibits the toxic effects caused by oxidative bursts by improving the ability of plants to scavenge ROS via the antioxidant defense system [24]. This results in reduced oxidative damage to tomato seeds under nematode stress and improved resistance to salt stress in broad bean leaves [25,26]. Additionally, the synergistic effects of JA and other plant hormones, such as ABA, SA, and ethylene (ETH), on ROS scavenging processes are essential for enhancing plant responses to abiotic stressors, thus providing a solid foundation for improving overall stress resistance in plants.

### 2.2. Signal Transduction Substances

#### 2.2.1. Ca^2+^

Ca^2+^ is an indispensable element for plant growth and development and plays a vital role in biological processes such as stress resistance. Research by Hou et al. indicated that exogenous calcium can significantly affect plant height, root length, biomass accumulation, and root structure in *Brassica napus*, especially the growth of relatively thick roots [27]. Calcium also acts as a second messenger involved in the transmission of stress signals in plants. Numerous studies have demonstrated that exogenous calcium helps plants eliminate ROS [28]. Typically, exogenous calcium stimulates ROS production and acts as a signal to activate antioxidant enzymes (e.g., peroxidase, POD, catalase, CAT, and superoxide dismutase, SOD) and the AsA-GSH cycle within plants, thereby preventing oxidative damage [29,30]. Additionally, Ca^2+^ can directly regulate the ROS scavenging system. ROD1 (RESISTANCE OF RICE TO DISEASES1), a Ca^2+^ sensor, can activate catalase to scavenge ROS directly [31]. The interconnection and interaction between the Ca^2+^ and ROS signaling systems are highly important for regulating cellular signals, responding to environmental stresses, maintaining cellular homeostasis, and regulating growth and development. As research progresses, many details and mechanisms need further elucidation.

#### 2.2.2. GABA

GABA is synthesized through the GABA shunt in plants and can act as a metabolite or signal under salt stress. The production of GABA promotes its attachment to the cell surface, triggering Ca^2+^ entry into cells and activating the GABA Transporter 1(GAT1) and Ca^2+^/CaM-dependent GAD activity, thereby increasing GABA generation, increasing photosynthesis and antioxidant capacity, reducing oxidative damage, and enhancing plant salt tolerance [32,33,34]. Exogenous GABA application can reduce ROS levels and improve crop photosynthesis and antioxidant enzyme activity to alleviate oxidative damage in lettuce, wheat seedlings, corn, and other crops [35,36,37]. Although the role of GABA in regulating ROS levels and mitigating environmental stress is known, the specific mechanisms remain unclear.

### 2.3. Relationships between Exogenous Substance Application and Improvements in Salt Tolerance, Yield, and Quality

The application of exogenous substances is an effective means to increase the salt tolerance of plants and improve crop yield and quality. Exogenously applied plant hormones, amino acids, antioxidants, and mineral elements increase the salt tolerance of plants by inducing osmotic regulatory substances, removing excessive ROS, and regulating membrane stability. In production, the use of exogenous substances improves plant quality and increases yield by supplementing nutrients, increasing the accumulation of photosynthetic products and inducing the accumulation of secondary metabolites(Table 1).

## 3. Role of Ion Transporters in Salt Stress Research

(1) Na^+^ Membrane Transporters

Under salt stress, plant cells regulate Na^+^ absorption and transport through multiple cation channels and transporters to maintain cytosolic Na^+^ homeostasis. The HKT transporter, located on the plasma membrane, exhibits Na^+^ and K^+^ transport activity, enhancing plant salt tolerance via Na^+^ efflux and increased K^+^ transport [47,48]. Son of sevenless homolog 1(SOS1), a Na^+^/H^+^ antiporter on the plasma membrane, is crucial for expelling cytosolic Na^+^ [49]. The HAK transporter family is involved primarily in K^+^ uptake and transport, yet certain members also demonstrate Na^+^ transport activity, such as OsHAK2 in rice [50,51]. Additionally, ligand-gated channels such as CNGCs and iGluRs contribute to regulating Na^+^ homeostasis [52]. Arabidopsis NHXs are capable of regulating the balance of Na^+^ and K^+^ ions, as well as facilitating their transport [53].

However, when the concentration of cytoplasmic Na^+^ increases, these transporters play a role in sequestering Na^+^ into the vacuole while promoting the accumulation of K^+^ in the vacuole. Currently, research on this aspect relies primarily on model organisms such as Arabidopsis and rice, whose genetic manipulation is relatively straightforward. However, different plants exhibit distinct salt stress response mechanisms, limiting the generalizability of these findings to other crop species.

(2) K^+^ Membrane Transporters

Maintaining K^+^/Na^+^ homeostasis, which relies on K^+^ absorption and distribution, is crucial for plants under salt stress [54,55]. The plant K^+^ transport system encompasses three channel families and three transporter families (KT/HAK/KUP, KEA, and Trk/HKT). The HAK transporter family, which is involved in high-affinity K^+^ transport, is essential for maintaining K/Na homeostasis and enhancing salt tolerance. For example, OsHAK5 and OsHAK21 in rice enhance salt tolerance by promoting K^+^ accumulation. The KEA (KEA4, 5, and 6) transporters play crucial roles in maintaining pH and K^+^ homeostasis in the intima and plastid [56,57,58,59]. Furthermore, the expression of these transporters may be associated with potassium deficiency, salt stress, and osmotic stress. HKT transporters maintain Na^+^/K^+^ homeostasis under both potassium deficiency and saline–alkaline conditions and are categorized into two types, both of which contribute to plant salt tolerance [60,61,62]. Furthermore, intracellular signaling pathways, such as Ca^2+^ signaling, ROS signaling, and plant hormone signaling, may regulate K^+^ transporters and channels under salt stress, but their specific mechanisms of interaction and impact remain to be explored.

(3) Ca^2+^ transporters

Ca^2+^ is a pivotal ion for plant growth, development, and responses to biotic and abiotic stresses. Under salt stress, plant cells respond to high-salt stimuli by increasing cytosolic Ca^2+^ concentrations, but excessive Ca^2+^ can be toxic [63]. To maintain calcium homeostasis, plant cells rely on diverse channel proteins to regulate Ca^2+^ influx and efflux. Influx systems include channels such as hyperosmolality-induced [Ca^2+^]i increase(OSCA), cyclic-nucleotide–gated channels(CNGC), two-pore channel(TPC), and ionotropic glutamate receptor(iGluRs.) For example, OSCA in Arabidopsis functions as an osmolarity stress sensor in response to salt stress [64,65]. CNGCs and GLRs are involved in regulating stomatal movement and calcium signaling [66]. Ca^2+^ efflux is achieved primarily through Ca^2+^-ATPase and Na^+^-Ca^2+^ exchangers (NCXs) [67,68]. The Ca^2+^ transporter in the vacuole has increased Ca^2+^-ATPase activity in salt-tolerant plants, thereby more efficiently transporting cytoplasmic Ca^2+^ to the vacuole [69,70]. These channel proteins regulate Ca^2+^ transport and distribution in response to the cellular environmental changes induced by salt stress, participating in cellular stress responses and maintaining ion balance. While the roles of calcium channel proteins under salt stress are only partially understood, further research is needed to elucidate their activation and inhibition mechanisms, interactions with other signaling pathways, and specific molecular mechanisms.

(4) Chloride Ion Transporters

**Figure 1:**
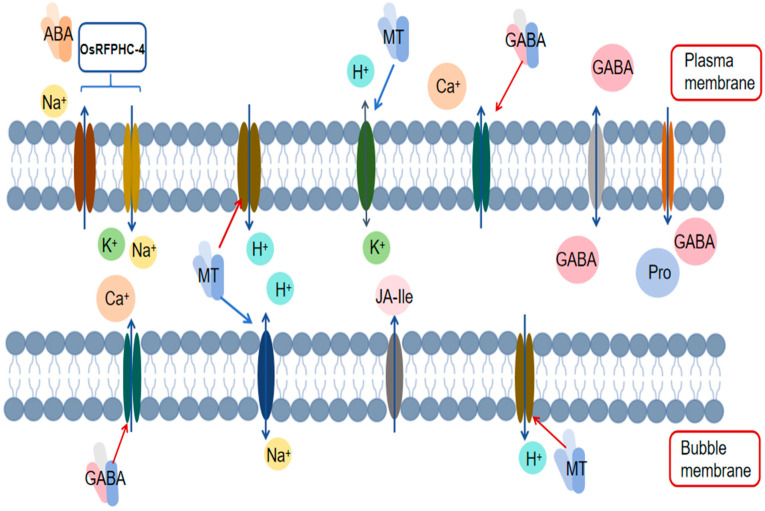
Mechanism by which exogenous substances regulate ion transporters.

Under salt stress conditions, maintaining the ion balance and cellular osmotic stability within plant cells is crucial for plant adaptation to high-salt environments. Chloride ion transporters located on the plant cell membrane participate in transmembrane ion transport, either directly or indirectly, during salt stress. Notably, the H^+^-ATPase on the plasma membrane generates a proton gradient by hydrolyzing ATP and pumping out H^+^, which provides the driving force for chloride ion efflux. Furthermore, the activity of H^+^-ATPase also facilitates the opening of anion channels, such as chloride channels (CLCs). In Arabidopsis, AtCLCc and AtCLCg are two chloride ion transporters localized within the cell membrane that exhibit high functional similarity and play a role in establishing plant salt tolerance [71,72]. Specifically, AtCLCc primarily participates in stomatal movement and salt stress regulation, whereas AtCLCg is localized to the tonoplast, where it contributes to maintaining cytoplasmic ion homeostasis by sequestering salt ions into the vacuole [73]. Additionally, the overexpression of *AVP1*, *PP2A-C5*, and *AtCLCc* has been shown to significantly increase plant tolerance to salt stress, drought stress, and their combined effects [74]. In addition to CLCs, slow anion channels (SLACs/SLAHs) also contribute to plant salt tolerance by regulating the transport of anions such as Cl^−^ and NO_3_^−^ in plant cells [75]. In summary, chloride ion transporters play pivotal roles in plant adaptation to salt stress, but further research is necessary to elucidate their specific mechanisms of action, regulatory networks, and impacts on plant salt tolerance (Figure 1).

## 4. Regulation of Ion Transport and Reactive Oxygen Species by Exogenous Substances

### 4.1. Plant Hormones Affecting Ion Transporters Involved in Regulating Reactive Oxygen Species Metabolism 

#### 4.1.1. Regulation of Ion Transport and Reactive Oxygen Species by ABA

The role of the interplay between ABA and ROS in plant growth and stress resistance is complex. There are many different mechanisms that link these substances together. Under stress, the rapid accumulation of ABA is involved in the regulation of gene transcription levels related to the ROS clearance system (ascorbate–glutathione cycle), which affects the redox state in plants. ABA inhibits ROS accumulation by increasing the activity of oxidases and key kinases in signaling pathways, as well as inducing stomatal movement [76]. OPEN stomata 1 (OST 1) is an important signaling element in the process of ABA-induced ROS generation that can phosphorylate the photosynthetic oxygen evolution protein PPD5 in chloroplasts, reduce ROS accumulation, and promote stomatal opening [77]. Plants overexpressing osrfphc-4 under high NaCl concentrations presented increased salt tolerance and increased ROS-scavenging enzyme activity; they also presented increased ABA sensitivity after exogenous ABA treatment. Furthermore, OsRFPHC-4 may improve plant salt tolerance by regulating Na^+^/K^+^ transporters, thereby maintaining cytoplasmic Na^+^/K^+^ homeostasis [78]. However, the specific molecular mechanisms by which it directly or indirectly affects ROS-scavenging enzyme activity and ABA signaling remain to be elucidated. ABI 4 is a key ABA-responsive transcription factor that enhances ABA signaling by inhibiting the expression of VTC 2, a key enzyme in the ascorbate biosynthesis pathway, and ABA-induced ROS generation, subsequently activating Ca^2+^ channels in the plasma membrane [76,79]. In this process, the role of ROS in the interplay between Ca^2+^ and ABA signaling remains to be elucidated.

#### 4.1.2. Regulation of Ion Transport and Reactive Oxygen Species by MT

MT, a novel plant hormone, can directly regulate ion channel activity. MT treatment enhances the H^+^ pump activity of H^+^-ATPase enzymes in tomatoes, endowing the enzymes with the ability to regulate ion homeostasis [80]. Regulating Na and K levels is crucial for improving salt stress recovery. Prolonged exposure to high-salt ion concentrations disrupts cytoplasmic Na, K, and Ca homeostasis, leading to the release of ROS. Studies have shown that exogenous MT enhances salt tolerance in sweet potatoes by increasing K uptake and Na levels [81]. During this process, key genes such as Na^+^/H^+^ antiporters (NHXs), K^+^ transporters (KATs), cation/H^+^ exchangers (CHXs), and CIPKs participate in managing ion homeostasis during salt stress, maintaining an optimal K/Na ratio within the plant [82]. Additionally, they facilitate Na^+^ sequestration into vacuoles, regulate stomatal movement, increase cytoplasmic Ca^2+^ concentrations, and maintain negative membrane potentials through elevated H^+^-ATPase activity. In addition to maintaining the ion balance, MT application enhances antioxidant enzyme activity and gene expression, as well as antioxidant accumulation, to maintain the ROS balance and prevent salt stress-induced membrane damage [83,84]. In addition, the melatonin-ROS-RNS signal can be used as a secondary messenger in response to stress. Melatonin induces H_2_O_2_ production by inhibiting the activity of mercaptonitrosylase and activating NADPH oxidase (RBOH), thereby regulating plant resistance to abiotic stress. H_2_O_2_/NO-melatonin-MAPK signaling plays an important role in plant immunity. MT induces the MAPK signaling cascade response by activating the kinase activity of MAPKKK3 and OXI1 (oxidative signal-inducible 1). As signal molecules, H_2_O_2_ and NO can play a certain role in the MT biosynthesis pathway by activating specific enzymes or inducing related genes. In contrast, MT can enhance the defense signal during pathogen infection via a positive feedback mechanism. The external application of MT can increase the cytoplasmic H_2_O_2_ level and then regulate the antioxidant enzyme system to protect plants from oxidative damage [85,86]. Although MT is widely regarded as a plant hormone, it can be an important secondary signal involved in the stress response. How does exogenous melatonin affect hormone content and signal changes through signaling pathways? How can ion channels be regulated directly or indirectly to control ROS? These questions need to be explored.

#### 4.1.3. Regulation of Ion Transport and Reactive Oxygen Species by JA

JA, an endogenous lipid hormone, has been widely studied in the context of plant biosynthesis [87]. Numerous studies have confirmed that JA and its metabolic derivatives play key roles in enhancing plant tolerance to abiotic stresses such as drought, heavy metal toxicity, and salt stress. These stress conditions affect metabolic pathways in plants by regulating gene expression and participating in ion transport in biofilms [88]. Upon stimulation by stress, cytosolic JA combines with ATP and isoleucine to form active Jasmonoyl-isoleucine(JA-Ile), which is transported to the nucleus by Jasmonatestransporter 1(JAT1) and binds to the COI1 protein in the SCF–COI1 complex. This interaction promotes the degradation of Jasmonate ZIM-domain(JAZ) proteins, releasing the inhibition of the transcription factor MYC2 and activating JA-responsive genes [89,90]. Takanori Maruta et al. reported that the AtrbohD and AtrbohF genes mediate ROS production and MeJA-responsive gene activation under Methyl jasmonate(MeJA) treatment. The ROS production mechanism mediated by Atrbohs depends on COI1, a key component of the JA signaling pathway [91]. Additionally, JA participates in ROS scavenging by regulating signal transduction pathways, antioxidant enzymes, and nonenzymatic antioxidants, as well as by interacting with other plant hormones [92]. In the fields of plant physiology and molecular biology, the specific mechanisms involved in JA-mediated signal transduction networks and ROS metabolism remain to be explored, especially in terms of how JA regulates ROS homeostasis via additional transporters. Future research should focus on JA-induced gene expression changes, protein modification events, and how these changes affect the function and regulation of ROS-related transporters. Such studies could provide molecular insights into plant adaptation and resistance to biotic and abiotic stresses.

#### 4.1.4. Regulation of Ion Transport and Reactive Oxygen Species by Other Substances

The potassium transporter OsHAK9 is involved in regulating seed germination under salt stress. Studies have shown that OsHAK9 restricts the efflux of K^+^ in germinating seeds to maintain the cytoplasmic K^+^/Na^+^ balance. Exogenous GA3 treatment under salt stress partially alleviates the downregulation of gibberellin 4 (GA4) levels caused by the disruption of OsHAK9 [93]. The salicylic acid receptor NPR1 (non-expressed form of PR protein 1) is the main regulatory protein of SA-dependent defense responses (Table 2). NPR1-mediated SA signaling controls the transport of Na^+^ from roots to shoots under salt stress; during this transport process, the activity of H^+^-ATPase in roots increases, and the concentration of K^+^ in shoots increases [94]. ETH is not only a plant stress hormone but also a signaling molecule that mediates important biological processes, including abiotic stress [95]. Multiple studies have shown that ETH regulates salt tolerance responses by modulating ROS generation and ROS scavenging mechanisms [96]. ETH positively regulates the transcription level of AtrbohF, and the ROS produced by AtrbohF play important roles in regulating Na^+^/K^+^ homeostasis. The specific mechanisms of plant hormone-mediated regulation of ROS metabolism involve various transporters that affect ROS production and scavenging through multiple mechanisms, thereby participating in plant adaptation to environmental stress.

### 4.2. Signaling Molecules Affecting Ion Transporters Involved in Regulating Reactive Oxygen Species Metabolism

#### 4.2.1. Regulation of Ion Transport and Reactive Oxygen Species by Ca^2+^

As an essential nutrient and a crucial secondary messenger, Ca^2+^ plays a pivotal role in plant development and stress response regulation through the involvement of calcium-dependent protein kinases (CDPKs, CPKs) and CBL-interacting protein kinases (CIPKs) [97]. In Arabidopsis, Ca^2+^ signaling is induced by cadmium stress, where CDPKs such as AtCPK21 and AtCPK23 function as positive regulators of Cd tolerance by inhibiting the Cd transport activity of the plasma membrane-localized transporter AtNramp6 through direct interaction and phosphorylation [98]. CPK/CDPKs serve as key regulatory proteins in plant stress signaling and are capable of binding Ca^2+^ and directly transmitting Ca^2+^ signals through their kinase domains [99]. They also participate in ROS regulation by phosphorylating various target proteins, including RBOH. Calcium plays a significant role in protecting plants from cadmium toxicity and regulating long-distance cadmium transport between plant tissues. In rice, H_2_O_2_ enhances Cd transport from roots to shoots by increasing *OsHMA2* expression to increase Cd xylem loading and downregulating *OsHMA3* to limit vacuolar sequestration of Cd. Exogenous Ca treatment further increased the Cd transport concentration under these conditions [100]. There is a complex interplay between Ca^2+^ signaling and ROS. Ca^2+^ and H_2_O_2_ cascades are involved in plant adaptive responses to environmental stresses. CDPKs regulate H_2_O_2_ production and scavenging by phosphorylating enzymes related to ROS metabolism. Additionally, the LRR receptor kinase HPCA1, located at the plasma membrane, acts as an H_2_O_2_ receptor, mediating H_2_O_2_-induced Ca^2+^ channel activation. This process regulates stomatal movement in response to environmental H_2_O_2_ levels, enabling plants to adapt to changing environments [101]. In addition, ROS, as signaling molecules, can respond to the response signals generated under environmental stress, and this process is regulated by RBOH activation mediated by Ca^2+^ or phosphorylation on the plasma membrane. On the plasma membrane, the regulation of RBOH by Ca^2+^, phosphorylation, and hormones can produce ROS in extracytosomes, which may act as signaling molecules to regulate the entry of aquaporins into the cytoplasm and jointly change the REDOX state of key regulatory proteins with metabolism- or signaling-related ROS produced in chloroplasts, mitochondria, and peroxisomes [31,102]. While Ca^2+^ can regulate ROS by modulating transporter activity via diverse mechanisms, research in this area is limited, potentially owing to the complexity of the underlying signaling networks.

#### 4.2.2. Regulation of Ion Transport and Reactive Oxygen Species by GABA

As an endogenous plant signaling molecule, GABA, owing to its structural flexibility, participates in regulating various physiological growth processes and biotic/abiotic stress responses. The intracellular transport of GABA is regulated by various transporters, including aluminium-activated malate transporters (ALMTs), GABA transporters (GATs), and proline transporters (ProTs) [103]. GABA negatively regulates anion efflux through ALMT channels, leading to changes in the cellular membrane potential through depolarization, which subsequently affects signal transduction. ALMTs are considered GATs with anion channel activity. Under acidic or alkaline conditions, TaALMT1-mediated GABA efflux is inhibited by malate and Al^3+^ [104]. Additionally, Al^3+^ can induce ROS production, causing oxidative stress, whereas exogenous GABA alleviates H^+^- and Al^3+^-induced ROS accumulation and protein and lipid damage [105]. GAT1 is a GABA transporter localized on the cell membrane. AtGAT1 has been confirmed to be a H^+^-driven, proton-coupled GABA transporter. Recently, CsGAT1, which has GABA transport activity, was identified in the tea plant genome, suggesting that GABA in roots may be transported to leaves by CsGAT1 under drought stress [106]. GABP, a mitochondrial GABA transporter, not only plays a crucial role in primary carbon metabolism by mediating GABA transport from the cytosol to the mitochondria, supporting normal plant growth under carbon limitation, but also may participate in the TCA cycle [107]. Yuan et al. recently echoed this viewpoint. ProTs were initially believed to transport only proline and not other protein amino acids [103]. However, Lin JH et al. demonstrated that plasma membrane-localized OsProT1 and OsProT3 specifically mediate the uptake of Pro and GABA into plant cells, albeit with low transport affinity. Furthermore, GABA metabolism has been shown to be related to Ca^2+^ signaling, where glutamate decarboxylase (GAD), which acts as a GABA receptor, binds to calmodulin (CaM) to regulate GABA metabolism [108]. GABA responds to environmental stress through the Ca^2+^/CaM-dependent protein kinase (CCaMK), which serves as a Ca^2+^ signal decoder. GABA activates certain types of Ca²⁺-ATPases and regulates cytosolic Ca²⁺ concentrations. Plasma membrane depolarization leads to the closure of voltage-gated Ca²⁺ channels, impeding Ca²⁺ influx and regulating cytosolic Ca²⁺ concentrations. These findings suggest that GABA inhibits ROS generation by maintaining cytosolic Ca²⁺ homeostasis. The precise mechanism by which GABA regulates transporters to eliminate ROS represents a complex regulatory process, the clarification of which requires further research. Future studies should focus on signal transduction pathways at the molecular level, the structural functions of transporters, and the integration of these processes at the overall plant level. This will allow elucidation of the regulatory mechanism of GABA in the maintenance of plant ROS homeostasis.

**Table 2:** Transporters involved in ROS signal regulation.

Name	Species	Description	Family
*NbPIP2;2* [109]	*N. benthamiana*	H_2_O_2_ transporter protein that guides extracellular H_2_O_2_ through the plasma membrane into cells and regulates plant immunity and ROS accumulation.	AQP
*AtPMTR1* [110]	*Arabidopsis*	Interacts with GPA 1, activates NADPH oxidase to produce H_2_O_2_, and promotes Ca^2+^ influx and K^+^ efflux to regulate stomatal motility.	GPCR
*NPR1* [111]	*Arabidopsis*	Accumulation of the SA receptor NPR 1 under salt stress reduces the accumulation of ROS in chloroplasts.	TF
*CsWAKL08* [112]	*Citrus reticulata Blanco*	SA- and JA-induced CsWAKL08 regulates plant resistance to pathogens by regulating ROS production and signaling.	RLK
*OsPM1* [113]	*Oryza sativa* L.	OsPM 1 serves as an ABA influx carrier to promote exogenous ABA introduction into cells and play a role in the response to drought stress and oxidative stress.	AWPM-19
*CRK2* [114]	*Arabidopsis*	The reciprocal regulation of CRK2 and RBOHD regulates ROS generation, stomatal movement, Ca^2+^ influx, and MAPK activation, which are important elements involved in immunity.	RLK
*SOS1* [115]	*Arabidopsis*	ROS acts as a signaling molecule to mediate SOS1 mRNA stability under salt stress, and SOS1 plays a key role in the regulation of NADPH oxidase activity and ROS generation.	NHX

## 5. Conclusions and Perspectives

**Figure 2:**
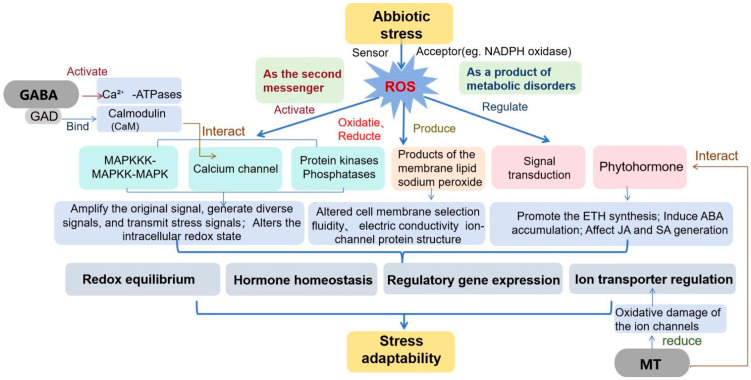
Mechanism by which exogenous substances regulate ROS affecting ion transport.

The response of plants to saline environments is a complex process involving morphological, physiological, biochemical, and genotypic changes [116]. These exogenous substances have numerous beneficial effects on plants under salt stress, including effects on signal transduction, energy supply, hormone induction, and antioxidant capacity [117]. In recent years, advancements in chemical synthesis and bioextraction techniques have significantly reduced the costs of various exogenous substances, such as amino acids, GABA, MeJA, and even MT. Moreover, these substances generally promote plant growth and increase yield and quality [39,45,46]. This makes the environmentally friendly application of exogenous substances more attractive, and these substances are expected to play a greater role in promoting the growth of halophilic plants and maintaining metabolic stability (Figure 2).

This article reviewed the effects of exogenous substances, such as plant hormones and signal transduction substances, on plant salt tolerance. During this process, ion transmembrane transport facilitates functions such as compartmentalization and replacement of harmful ions and participates in the regulation of ROS levels. Although ROS play an important role in regulating Na^+^/K^+^ homeostasis, this process involves multiple regulatory mechanisms; however, there is still limited evidence of a direct relationship between the two in existing research reports, and the mechanism remains unclear [118]. Therefore, it is necessary for the mechanisms of key ion transporters and ROS metabolism to be elucidated in future research.

## Figures and Tables

**Table 1:** Regulation of salt tolerance, quality, and yield by the application of exogenous substances.

Exogenous Additive	Varieties	Impact on Yield and Quality	
GA_3_	S79 and S80	Root length, root number, root weight, seedling weight, and growth rate increased by 12.82%, 9.45%, 17.84%, 7.06%, 36.73%, and 36.73%.	Hu W et al. [38]
CGG (GABA, CaCl_2_ and Glu compound)	Japonica rice: Dongnong 428, Songjing 10	Under the optimal concentration ratio (1.87 mmol/L CaCl_2_, 2.76 mg/mL Glu, 4.40 mmol/L GABA), the annual output of Dongnong 428 was 8340.5 kg/ha and that of Songjing 10 was 7093.5 kg/ha, which was 11.05% and 24.47% higher than the values observed under cold water stress treatment, respectively.	Jia Y et al. [39]
SNP, SA	Wheat: “Shanon 22”,	The yield of wheat treated with SA, SNP, slow-release SA, and SNP under salt stress increased by 16.35%, 9.62%, 22.12%, and 32.69%, respectively; the chlorophyll content in the seedling leaves increased by 5.51%, 7.85%, 12.98%, and 16.83%, respectively; and the O_2_^−^ content decreased by 26.78%, 34.58%, 27.65% and 39.06%, respectively, compared with CK.	Dong YJ et al. [40]
Salicylic acid (SA), calcium chloride (CaCl_2_), and abscisic acid (ABA)	Peony (*P. ostii* ‘*Fengdan*’)	Treatments with 100 μmol/L SA, 40 mmol/L CaCl_2_, and 240 mg/L ABA could all alleviate high-temperature damage. Taking soluble protein (SPs) as an example, the SP content of SA-treated seedlings increased by 34.00%, 17.67% and 19.29% at 2, 4 and 6 d, respectively; that of CaCl_2_-treated seedlings increased by 15.31%, 20.03% and 41.40% at 4, 6 and 7 d, respectively; and that of ABA-treated seedlings increased by 26.36%, 12.66%, 15.23% and 35.89% at 2, 4, 6 and 7 d, respectively, during the recovery period (all are compared with HT treatment).	Guo JX et al. [41]
Abscisic acid (ABA)	*Sauvignon* (*Vitis vinifera* cv. *Cabernet*)	Exogenous ABA treatment speeds up grape coloration. Colouration after ABA treatment started 5 days earlier than that in the CK group, with 10% coloration occurring per day 7 days after treatment (66 DAF) and full coloration observed at 72 DAF. Under carbon-limited ABA treatment, grapes changed color faster, at a 5.8% daily change rate, at 69 DAF compared to CK (3.3% daily).	Tong Q et al. [42]
GA_3_	Strawberry (*Fragaria* × *ananassa* Duch.)	The maximum fruit weight of 100 mgL^−1^ GA_3_-sprayed plants increased by 34.82%, the longitudinal diameter increased by 33.56%, and the total output increased by 54.84%. The yield of marketable fruit in this treatment group increased by 74.47% compared with CK, and the yield of nonmarketable fruit decreased by 14.64%.	Rathod K.D. et al. [43]
GA_3_	Kiwifruit (*Actinidia deliciosa* Chev.)	After 25 mgL^−1^ GA_3_ treatment, the leaf area in the first and second years increased by 11.54% and 8.48% over CK, respectively; the fruit weight increased by 30.51% and 31.43%, respectively; and the fruit hardness increased by 10.70% and 7.24%, respectively.	Nowsheen Nazir et al. [44]
Melatonin (MT)	Sunshine rose (*Vitis labrusca* × *vinifera* ‘Shine Muscat’)	Treatment with 150 μmol/LMT increased the yield by 8.28%, 8.20%, and 8.54%, and treatment with 50 μmol/LMT increased the yield by 4.41%, 4.09%, and 3.09%.	Xu N et al. [45]
H_2_S, GABA	Persimmon (*Diospyros kaki*)	Under 3 mM NaHS and 7.5 mM GABA treatment, persimmon peel had a low browning rate and more antioxidants (AsA, flavonoids), higher polygalacturonase (PG) and pectin methylesterase (PME) activity, and better hardness, DPPH clearance activity and membrane integrity under cold storage, allowing maintenance of better food quality.	Zohreh Niazi et al. [46]
